# Mass production of metal-doped graphene from the agriculture waste of *Quercus ilex* leaves for supercapacitors: inclusive DFT study

**DOI:** 10.1039/d0ra09393a

**Published:** 2021-03-15

**Authors:** Gaurav Tatrari, Chetna Tewari, Manoj Karakoti, Mayank Pathak, Ritu Jangra, Boddepalli Santhibhushan, Suman Mahendia, Nanda Gopal Sahoo

**Affiliations:** PRS-NSNT Centre, Department of Chemistry, D.S.B. Campus, Kumaun University Nainital 263001 Uttarakhand India ngsahoo@yahoo.co.in; Department of Physics, Kurukshetra University Kurukshetra India; Department of Electrical Engineering, Indian Institute of Technology Bombay 400076 Maharashtra India

## Abstract

This work reports a facile, eco-friendly, and cost-effective mass-scale synthesis of metal-doped graphene sheets (MDGs) using agriculture waste of *Quercus ilex* leaves for supercapacitor applications. A single step-degradation catalyst-based pyrolysis route was used for the manufacture of MDGs. Obtained MDGs were further evaluated *via* advanced spectroscopy and microscopic techniques including Raman spectroscopy, FT-IR, XRD, SEM/EDX, and TEM imaging. The Raman spectrum showed D and G bands at 1300 cm^−1^ and 1590 cm^−1^, respectively, followed by a 2D band at 2770 cm^−1^, which confirmed the synthesis of few-layered MDGs. The SEM/EDX data confirmed the presence of 6.15%, 3.17%, and 2.36% of potassium, calcium and magnesium in the obtained MDGs, respectively. Additionally, the FT-IR, XRD, TEM, and SEM data including the plot profile diagrams confirmed the synthesis of MDGs. Further, a computational study was performed for the structural validation of MDGs using Gaussian 09. The density functional theory (DFT) results showed a chemisorption/decoration pattern of doping for metal ions on the few-layered graphene nanosheets, rather than a substitutional pattern. Further, resulting MDGs were used as an active material for the fabrication of a supercapacitor electrode using the polymer gel of PVA–H_3_PO_4_ as the electrolyte. The fabricated device showed a decent specific capacitance of 18.2 F g^−1^ at a scan rate of 5 mV s^−1^ with a power density of 1000 W kg^−1^ at 5 A g^−1^.

## Abstract 2

This work reports a facile, eco-friendly, and cost-effective mass-scale synthesis of metal-doped graphene sheets (MDGs) using agriculture waste of *Quercus ilex* leaves for supercapacitor applications.

## Graphical abstract

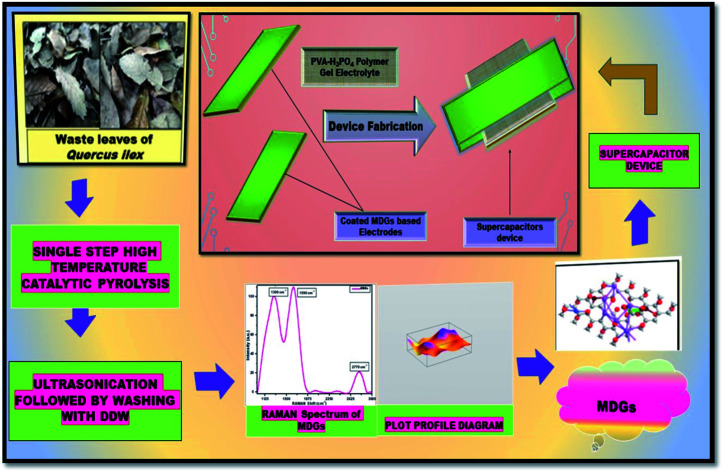

## 1. Introduction

The increase in agriculture and forest waste is not only the major cause for nearly every forest fire, destruction of forests, and burning of wild animals, but also produces greenhouse gases (GHG), which have a direct effect on the rising global temperature and air pollution. It is also the major cause for the disrupted growth of various traditional therapeutic medicinal plants, timbers, fruits and nuts, vegetables, resins, essences barks, and fibers such as bamboo, rattans and other related resources. A recent study revealed that over 25% of the population depends on these ecological resources for their living.^1,2^ Accordingly, the sustainable management of agriculture waste (AGW) through greener pathways and their economic conversion into value-added advanced products such as graphene is one of the biggest tasks for the scientific society. Pyrolysis technology with the valued combination of nanoscience seems to be a suitable strategy to deal with this problem. The production of graphene nanosheets through the pyrolysis approach in conjunction with nanoscience is not only a cost-effective process, but also does not produce serious environmental hazards, and thus can boost waste management technologies to the next level. Graphene is composed of 2D single-layered sheets of graphite having sp^2^ hybridization. Recently, graphene has emerged as a new potential candidate for direct application in various fields of science and technology, *i.e.*, energy conversion and energy storage devices, bio-imaging, drug delivery, fuel cells, and biosensors.^2–5^ This is mainly due to the marvelous properties of graphene such as its high electrical conductivity, huge surface area, light-weight structure, and excellent mechanical and tensile strength.^6,7^ In addition, metal-doping in graphene nanosheets enhances their potential application, especially in the field of energy storage and conversion devices, fuel cells, polymer composites, and bio-sensing applications.^6–8^ Previously, various methods have been introduced for the qualitative production of metal ion-doped graphene nanosheets *via* physical vapor deposition (PVD), chemical vapor deposition (CVD), coupling reactions, electrochemical exfoliation, and Hummers method along with the solvolytic approach.^8^ However, the mass production of metal-doped graphene nanosheets using an eco-friendly and cost-effective route is still a challenge in the scientific community. AGW is a good option, which can be utilized as a raw material for the production of metal-doped graphene nanosheets (MDGs). The mass production of MDGs using AGW as a precursor not only acts as a complete solution to clean up the ecological problems created by AGW, but also provides a new pathway for the mass recapture of AGW and the production of value-added products such as MDGs. Various studies have been reported for the qualitative production of carbon nanomaterials from solid waste materials, *i.e.*, from plastic waste, rice husk, sugarcane bagasse, coconut shells, coffee beans, and bio-materials.^9–11^ Different researchers have reported that polypropylene mixed organically-modified montmorillonite clay is an excellent agent for the production of high-quality nanomaterials from carbonaceous materials.^11,12^ Some other studies revealed the excellent catalytic potential of bentonite clay and ZSM-5 for the conversion of carbonic solids into carbon nanomaterials (CNMs).^12–15^ Thus, the use of clay such as bentonite and ZSM-5 for the conversion of AGW into graphene nanosheets using high temperature pyrolysis is an extremely cost-effective, environment friendly, and green approach for the synthesis of graphene sheets. Various attempts have been made by researchers to produce graphene oxide (oxidized form of graphene); however, little progress has been made in the bulk production of metal-doped graphene nanosheets starting from AGW.^13–16^

Herein, we report the mass-scale transformation of a waste material, *i.e.*, *Quercus ilex* leaves, into MDGs using a cost-effective, eco-friendly, and green pathway. The synthetic route includes a simple single-step pyrolysis using a mixture of ZSM-5 and bentonite clay as the degradation catalysts. The high-temperature pyrolysis approach was followed for the appropriate degradation of carbon sheets into MDGs. However, the catalyst used is much cheaper, easily available, and has almost zero environmental toxicity compared to the reported methods. In addition, a theoretical DFT study was performed to reveal the appropriate incorporation pattern of metallic ions over the graphene nanosheets and determine the structural details of MDGs. The obtained MDGs were further utilized applied in the fabrication of a supercapacitor with PVA–H_3_PO_4_ polymer gel electrolyte, resulting in the good efficiency of the fabricated device. The present study acts as an extremely useful method for the mass-scale high-quality production of MDGs and also reveals an economically viable synthetic approach to preserve our ecology and environment. Furthermore, the supercapacitor behavior of the synthesized MDGs in the polymer-gel electrolyte (PVA–H_3_PO_4_) showed good efficiency, which is another benefit for the sophisticated management of agricultural waste. The fabricated device showed a good specific capacitance of 18.2 F g^−1^ with a power density of 1000 W kg^−1^ and excellent energy density of 2.5 W h kg^−1^ at 5 A g^−1^, which can be very useful for futuristic energy storage devices. Hence, this study widens the scope of waste management in an applicative manner and can motivate research in the direction of “waste to wealth”.

## 2. Materials and methods

### 2.1. Materials

*Quercus ilex* leaves were collected from a nearby forest of the Nainital region situated in Uttarakhand, India. Bentonite clay, ZSM-5 and solvents used during the experiments were purchased from Aldrich and were used as received. In addition, phosphoric acid (H_3_PO_4_) was purchased from Sigma Aldrich and PVA was purchased from Laboratory Rasayan, India, which were both used as received.

### 2.2. Synthesis of MDGs

The synthesis of MDGs was performed using a single-step pyrolysis approach. In brief, firstly *Quercus ilex* leaves were collected from a nearby forest and the collected leaves were sun-dried for a day, and then washed with a diluted ordinary soap solution followed by double distilled water several times. Then the leaves were further dried and chopped into fine pieces. The chopped leaves were weighed and found to be about 5 kg and further processed in the pyrolysis unit by mixing with 2% degradation catalyst, *i.e.*, a mixture of ZSM-5 and bentonite clay in a 1 : 1 ratio. Then the furnace temperature was increased to 820 °C at a constant heating rate of 5 °C min^−1^. Then the process was held at the temperature of 820 °C for the next 1.5 h to maintain the temperature for the degradation and exfoliation reaction. The high temperature and presence of degradation catalysts resulted in the excellent decomposition of the carbonic structure. Usually, higher layers of carbon degrade into smaller units of carbonic allotropes, which were further exfoliated by maintaining the same condition for the next 1.5 h. After the completion of pyrolysis, the resulting material was collected and treated with dilute H_2_SO_4_ for 1 h in a magnetic stirrer at a continuous speed, which oxidized the vacant sites into oxidizing functionalities. The MDG sample was further processed in an ultrasonicator for 1 h using dilute HCl, which uses sound waves to perturb the solution, assisting the segregation of impurities. Further, the final washing process was done with double distilled water until the solution became neutral. This process was necessary for the removal of impurities from the MDGs. Finally, the obtained MDG sample was dried, collected, and weighed to be about 2.0 kg in the form of a fine powder.

### 2.3. Preparation of gel electrolyte

The gel electrolyte was prepared by mixing polyvinyl alcohol (PVA), double distilled water (DDW), and phosphoric acid (H_3_PO_4_) using a simple solvothermal approach. In brief, 1 g of PVA was added to hot DDW and continuously stirred for 40 min at 95 °C until a transparent and homogeneous mixture was obtained. Further, 1 g of H_3_PO_4_ was slowly added to the solution of PVA–DW and stirred for 15 min for the evaporation of water to obtain a thin film of gel electrolyte (PVA–H_3_PO_4_).^17–20^

### 2.4. Device fabrication

A device was fabricated using 90% (wt%) slurry of MDG and 10% (wt%) polyvinylidene fluoride (PVDF) as the binder. In brief, 10 wt% PVDF powder was dissolved in acetone through constant stirring for 6 h in an enclosed beaker to restrict the evaporation of the solvent. Subsequently, 10 wt% PVDF solution was slowly mixed with 90 wt% MDG material to prepare a slurry using a mortar and pestle.

**Scheme 1:**
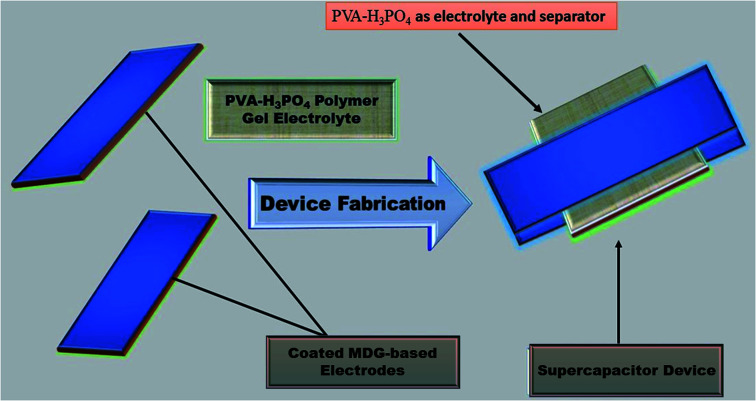
Fabrication of MDGs-based supercapacitors.

Further, a slurry of MDG (1 mg) sample was coated on two symmetrical graphite sheets with an area of 1 × 1 cm^2^. The prepared MDG-coated graphite sheets (electrode) were further left in an oven overnight at 90 °C. Then, the prepared film of polymer gel electrolyte (1 × 1 cm^2^ area) was placed between two MDG-treated electrodes, resulting a structure similar to a sandwich (Scheme 1). Here, the PVA–H_3_PO_4_ polymer gel electrolyte play two concurrent roles as the electrolyte and separator in the fabricated devices (Table 1).

**Table 1:** Cell architecture with MDGs over graphite sheets as the current collector

S. no.	Cell structure
1	Graphite sheets|GNs|PVA–H_3_PO_4_|GNs|graphite sheets

### 2.5. Material characterization

To confirm the successful synthesis of MDGs, various advanced spectroscopic and microscopic characterization techniques were performed. Raman spectroscopy (RIRM-LPI519) with an excitation beam wavelength of 532 nm was used to identify the D, G and 2D peaks and related properties of the synthesized MDGs. A PerkinElmer Spectrum-2 FT-IR spectrometer was used for the accurate detection of the chemical functionalities in MDGs. Further, MDGs were evaluated using X-ray diffraction spectroscopy (XRD) on a Rigaku MiniFlex-II spectrometer having Cu-Kα radiation with a wavelength of 1.54 Å. A Carl Zeiss Supra 55 scanning electron microscope (SEM) was used for the detection of the external morphology of MDGs. The internal morphology of the MDG sample was detected using a JOEL JEM 2100 Plus, tunneling electron microscope (TEM). Electron dispersive X-ray (EDX) analysis was carried for the detection of the exact elemental and metallic composition of MDGs. A theoretical computational DFT study was also performed for the precise validation of the structural bonding between the metals and graphene sheets of the synthesized MDG.

### 2.6. Device characterization

The electrochemical performance testing of the MDG-based device was done using an electrochemical workstation (CHI 660E, CH Instruments, Inc.). Cyclic voltammetry (CV) was performed to measure the specific capacitance using a two-electrode system. CV was performed at a scan rate in the range of 5 mV s^−1^ to 500 mV s^−1^ in the potential window of −1.0 to +1.0 V in PVA–H_3_PO_4_ polymer gel electrolyte. Electrochemical impedance spectroscopy (EIS) was performed at 10 mHz, while charge/discharge was carried out at a current density of 1, 2 and 5 mA cm^−2^ in the potential window of 0–1 V. Additionally, the specific capacitance was calculated using the CV, electrochemical impedance spectroscopy (EIS) and galvanostatic charge–discharge (GCD) techniques.

## 3. Results and discussion

### 3.1. Material characterization and DFT evaluations

Several advanced spectroscopic and microscopic techniques were used for the structural and chemical evaluation of MDGs, among which Raman spectroscopy is one often regarded as the most prominent technique for the structural identification of graphene and its counterparts, *i.e.*, graphene oxide, carbon nanotubes (CNTs), and graphite. The Raman spectroscopical details revealed some vibrational in-plane and out-of-plane active modes for the corresponding carbonic framework, which specifically showed D and G bands at 1300 cm^−1^ and 1590 cm^−1^ respectively (Fig. 2). The intensity ratio of the D and G band, *i.e.*, *I*_D_/*I*_G_ was found to be 0.82. Together with this, the Raman spectrum also depicts a well-developed 2D peak at 2770 cm^−1^. In detail, the D band represents the deformation, which occurs due to the conversion of the sp^2^-hybridized carbon atoms of the graphitic structure into sp^3^-hybridized carbon atoms of graphene sheets.

**Fig. 1:**
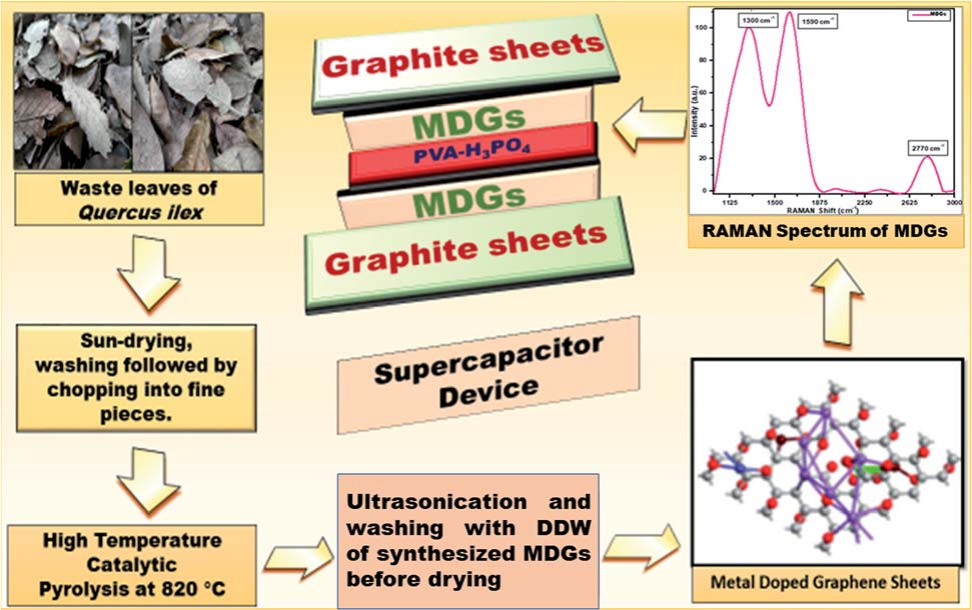
Schematic diagram of the complete process.

The G band in the Raman spectrum shows the well-established sp^2^-hybridized carbon atoms of the graphene sheets. Further, the 2D band confirms the presence of a graphene-based structure.^20–22^ The ratio of the *I*_D_/*I*_G_ peak intensity is a relative measure of all the defects existing on MDGs. The appearance of the D peak shows presence of defective MDGs. These (*I*_D_ and *I*_G_) peaks are the result of the vibrations of sp^2^ carbon atoms. Conversely, the G peak is a relative measure of the in-plane vibrations of the sp^2^ carbon atoms, and the D peak is mainly due to the out-plane vibrations ascribed to the structural defects.^22,23^ The *I*_D_/*I*_G_ ratio is related to the sp^3^/sp^2^ carbon ratio if the carbon material is oxidized, in other words, the MDG carbon atoms are sp^3^ hybridized. A higher D peak represents broken sp^2^ bonds, which indicates presence of more sp^3^ bonds, and thus a higher the transition from sp^2^ to sp^3^ in MDGs, resulting in the maximum D/G ratio. A high *I*_D_/*I*_G_ ratio represents the presence of defects on MDGs, whereas a low *I*_D_/*I*_G_ ratio of the carbon material shows graphitization in MDGs. The *I*_D_/*I*_G_ ratio that found to be 0.82, while the *I*_2D_/*I*_G_ ratio was found to be 1.76. Monolayer graphene usually shows an *I*_D_ of zero, and *I*_2D_/*I*_G_ of 0.5. However, the CNM sample with structural disorder represents multilayer graphene. Thus, the *I*_D_/*I*_G_ ratio and *I*_2D_/*I*_G_ ratio indicate the presence of few-layer graphene nanosheets.^21,22^ Further, the XRD spectrum of the obtained material can be seen in Fig. 1, which shows two preferential broad peaks of graphene at 2*θ* = 24° and another peak at 2*θ* = 43°. These characteristic XRD peaks correspond to the presence of graphene nanosheets, and also strengthen above-discussed Raman spectrum.^23,24^

**Fig. 2:**
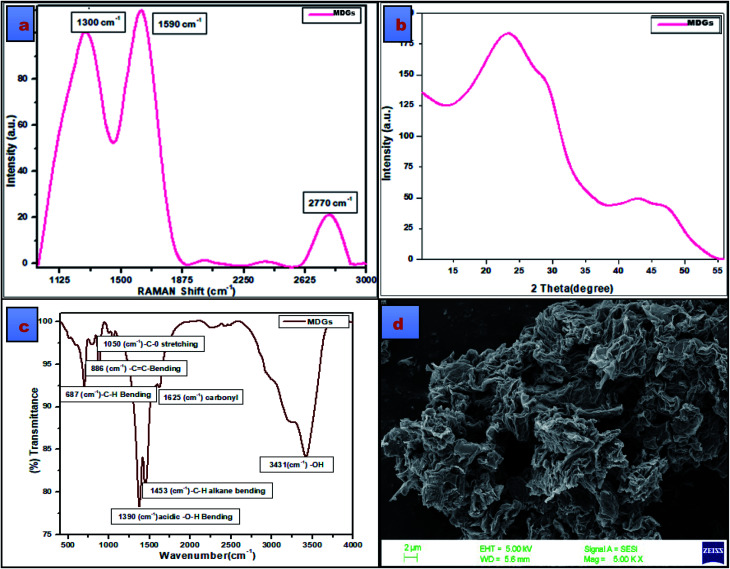
(a) Raman spectrum of MDGs, (b) XRD pattern of MDGs, (c) FT-IR spectrum of MDGs and (d) SEM image of MDGs.

To further confirm the presence of functional moieties in MDGs, their FT-IR spectrum was evaluated. The evaluation of the FT-IR data showed the presence of underdeveloped peaks at 687 cm^−1^, 806 cm^−1^, 1050 cm^−1^, and 1625 cm^−1^, corresponding to the C–H bending vibration, C–C bending vibration, C–O stretching vibration, and carbonyl stretching vibration, respectively. The peaks at 1390 cm^−1^, 1455 cm^−1^, and 3431 cm^−1^ correspond to the –OH bending vibration, C–H bending vibration of alkane, and –OH stretching vibrations, respectively. Thus, the FT-IR data showed the presence of some oxidizing functional groups in MDGs. The presence of underdeveloped oxidizing functional groups indicates the partial oxidation of the graphene sheets, which may be due to the acidic treatment during their synthesis. The FT-IR data is well supported by the SEM microscopic analysis, which was used for the identification of the surface morphology of the developed sheets. The SEM image showed a stacked layered fashion arrangement of MDGs.

**Fig. 3:**
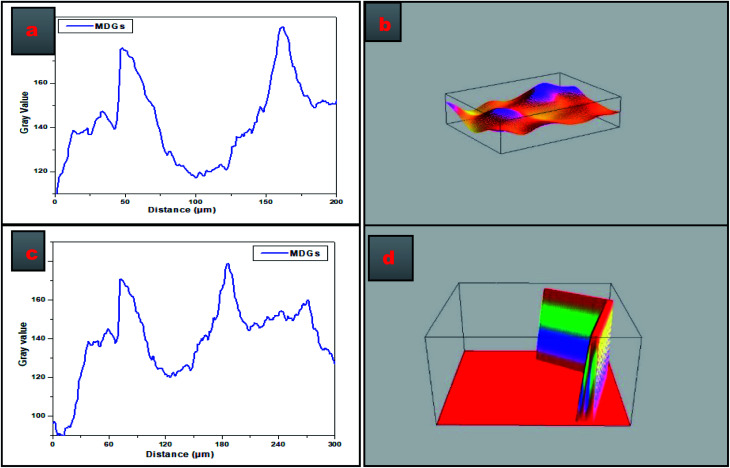
(a–d) SEM plot profiles of MDGs.

Fig. 3 presents the SEM image-based plot profiles of MDGs, which were used for the analysis of the surface morphology of the synthesized MDGs, where Fig. 3a and c depict the graph of the gray value as a function of distance in μm for the critical analysis of randomly selected parts of the SEM images of MDGs. The plot profile diagram shows that MDGs consist of stacked layers having a thickness of 0.2–0.6 μm. Fig. 3b and d show the 3D surface morphology of randomly selected MDGs obtained by plot profile evaluations for randomly selected portions of MDGs. Fig. 3b shows the horizontally aligned morphology of the SEM image of MDGs, in which the orange-yellow contrast shows the planner arrangement of the MDG layers. Fig. 3d shows the vertically aligned arrangement of MDGs for a randomly selected area of the SEM image. The uneven pattern of the plot profile may be due to the incomplete reduction of MDGs at a higher exfoliation temperature.

**Fig. 4:**
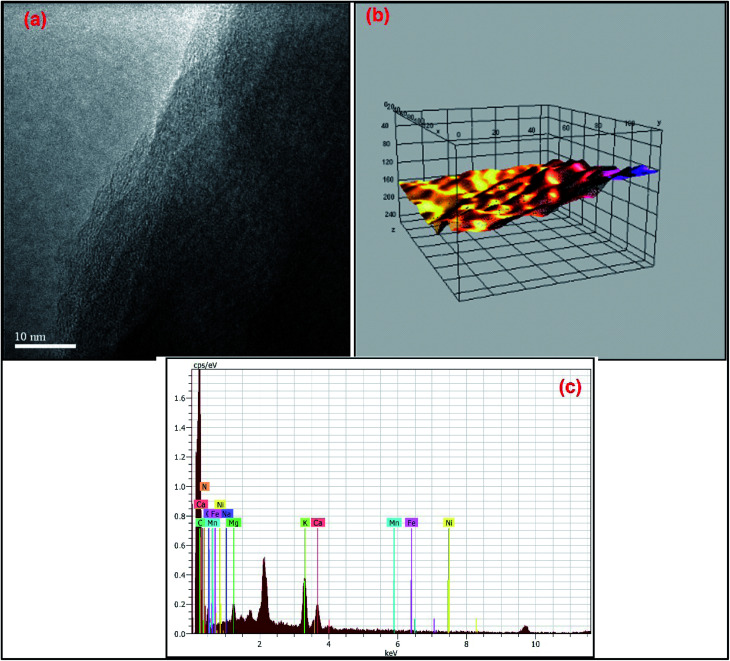
(a) TEM image with 10 nm resolution of MDGs, (b) plot profile diagram of MDGs and (c) EDX spectrum of MDGs.

Additionally, TEM imaging was done to evaluate the internal morphology of MDGs. The TEM image (Fig. 4a) clearly showed the *n* < 5 (where *n* is the number of layers) layered arrangement of MDGs. The plot profile diagram of a randomly selected portion of the TEM image showed the excellent linearly arranged layers, where the yellow contour indicates the incomplete sites of reduction at high temperature exfoliation. Further, for the evaluation of the presence of metals in MDGs, we conducted an EDX analysis, which is well recognized as a basic tool for the detection of chemical composition.

The EDX corresponding data indicated 6.15% of potassium together with 3.17% of calcium and 2.36% magnesium by atomic weight percentage as the major metals (Fig. 4b and Table 2). Thus, the EDX data depicts the clear presence of potassium in the obtained graphene sheets as the naturally doped metal ion.

**Table 2:** EDX spectrum-based elemental composition details of MDGs

Element	AN series	UNN.·C [wt%]	Norm. C [wt%]	Atom. [at%]	Error (1 sigma) [wt%]
C 6	K-series	9.24	41.48	56.30	3.44
O 8	K-series	6.67	29.96	30.52	3.62
**K 19**	**K-series**	**3.28**	**14.74**	**6.15**	**0.24**
**Ca 20**	**K-series**	**1.74**	**7.79**	**3.17**	**0.18**
Mg 12	K-series	0.78	3.52	2.36	0.13
Na 11	K-series	0.41	1.83	1.30	0.11
Mn 25	K-series	0.15	0.69	0.20	0.08
Fe 26	K-series	0.00	0.00	0.00	0.00
Ni 28	K-series	0.00	0.00	0.00	0.00
N 7	K-series	0.00	0.00	0.00	0.00

Further, to understand the positioning of the dopants in the graphene sheet, density functional theory (DFT)-based first-principles simulations were performed as implemented in the Synopsys-QuantumATK code.^22–26^ The exchange–correlation interaction energy of the electrons was described by the Perdew–Burke–Ernzerhof (PBE) functional within the generalized gradient approximation (GGA). The semi-empirical Grimme DFT-D2 (ref. 27) correction was used to include the van der Waals interactions between the dopant atoms and the graphene sheet. The localized pseudoatomic orbitals with double zeta polarized basis sets were utilized to describe the valence electrons. A large density mesh cutoff of 130 hartree was considered for the accuracy of the calculations. The Brillouin zone of 5 × 5 and 7 × 7 hexagonal supercells of MDGs was sampled with a Monkhorst–Pack grid of 10 × 10 × 1 and 7 × 7 × 1 *k*-points, respectively. A large vacuum gap of 25 Å was maintained in the out-of-plane direction of MDGs to avoid interactions among the periodic images. The structural relaxations were performed with the help of the limited-memory Broyden–Fletcher–Goldfarb–Shanno (L-BFGS) quasi-Newton method^25–28^ so that the forces on the atoms and stress on the supercell converge below the tolerance value of 0.05 eV Å^−1^ and 0.0006 eV Å^3^, respectively.

**Fig. 5:**
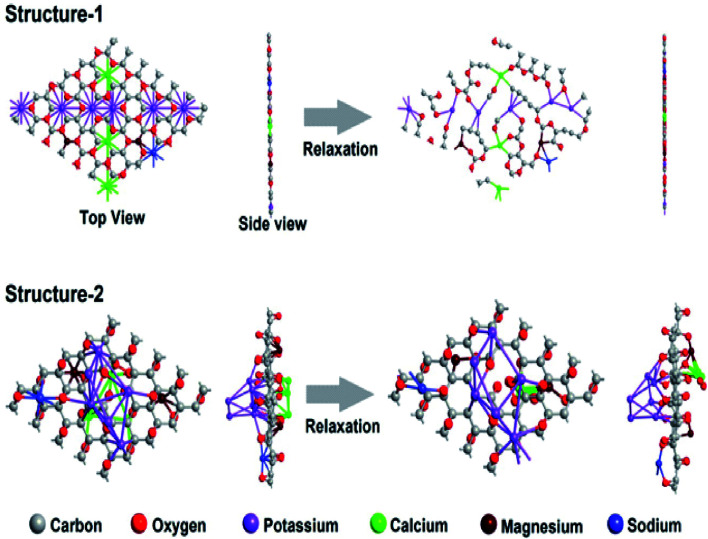
Structures of metal-doped graphene (MDG) before and after relaxation. In structure-1 the doping was done by substituting the carbon atoms and in structure-2 doping was done through chemisorption/decoration of the dopants over the carbon atoms.

The size of these supercells was chosen in such a way that the number of atoms inside the supercell was below 100 for computational feasibility, and the atomic composition was comparable to the experimental samples (see Table 3). As seen in Fig. 5, structure-1 lost its shape and shattered into pieces after relaxation, whereas, structure-2 retained its shape and appeared to be stable after relaxation. This indicates that the positioning of the dopants in our heavily doped experimental MDG samples may follow the chemisorption/decoration pattern on the sheet, rather than the substitutional pattern. Thus, the chemisorption pattern of decoration evaluated by the DFT study confirms the presence of van der Waals interactions between the dopant atoms, *i.e.*, metal ions, and the graphene sheets in MDGs. This may be due to the interaction between the potassium ions and presence of unsaturation on the carbon surface sites of MDGs. However, the broadness advance modeling of the layered MDGs through *ab initio* molecular dynamics, and DFT/synthetic growth concept also confirm the presence of the chemisorption/decoration pattern of the dopant on MDGs.^27–30^

**Table 3:** The atomic composition in the experimental samples and computational structures

Atom type	Atomic composition (%)		
	Experimental	Structure-1	Structure-2
Carbon	56.30	57.14	55.68
Oxygen	30.52	30.61	30.33
Potassium	6.15	6.12	6.74
Calcium	3.17	3.06	3.37
Magnesium	2.36	2.04	2.24
Sodium	1.30	1.02	1.12
Manganese	0.20	—	—

### 3.2. CV analysis

Cyclic voltammetry was conducted in a two-electrode cell system for the MDG-based fabricated device at different scan rates. Here, PVA–H_3_PO_4_ was used as the gel electrolyte, which acted as a separator and electrolyte in the processes of the cell cycle. The current (A) *vs.* voltage (V) plot of the CV data showed a relative square-shaped curve, which predicts the ionic interaction mechanism of the electrode and gel electrolyte.^18,31^ In brief, the square-shaped curve predicts the formation of a double layer at the interface of the electrodes and electrolyte, which is usually the result of achieving ionic separation together with a Helmholtz double layer. The specific capacitance was calculated using eqn (1). The fabricated device showed a good specific capacitance of 18.2 F g^−1^ at the scan rate of 5 mV s^−1^ (Table 4). Furthermore, the range of specific capacitance for the fabricated devices was relatively stable with a further increase in scan rate, as shown in Table 4.1
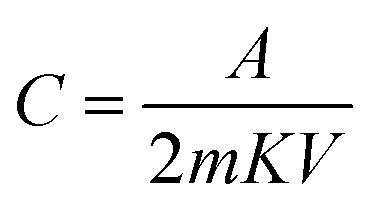
where *A* is the integrated area of the CV curve, *m* is mass in mg, *K* is the scan rate in mV s^−1^, and *V* is the potential in volts.

**Table 4:** The CV-based specific capacitance values of the fabricated device

Scan rate	5 (mV s^−1^)	10 (mV s^−1^)	20 (mV s^−1^)	50 (mV s^−1^)	100 (mV s^−1^)	200 (mV s^−1^)	500 (mV s^−1^)
Specific capacitance using CV (in F g^−1^)	18.2	16.25	15.26	14.2	12.6	10.9	8.65

Further, the energy density (*E*_D_) was measured using eqn (2) as follows:2
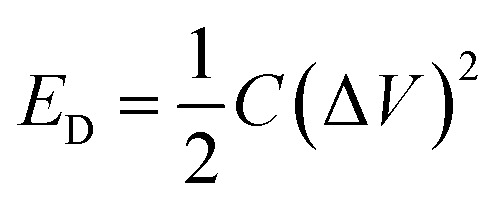
where *C* is the specific capacitance of the CV and Δ*V* is the operating voltage. The energy density of 2.5 W h kg^−1^ was obtained at 5 A g^−1^.

The power density (*P*_D_) was measured using eqn (3) as follows:3
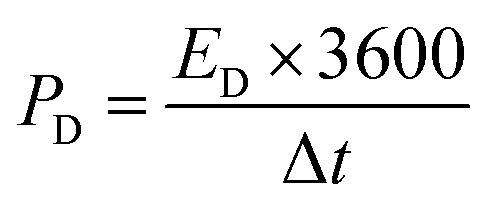
where *E*_D_ is the energy density and Δ*t* the time window during the potential range. The excellent power density of 1000 W kg^−1^ was measured for the fabricated device at 5 A g^−1^.

### 3.3. GCD analysis

**Fig. 6:**
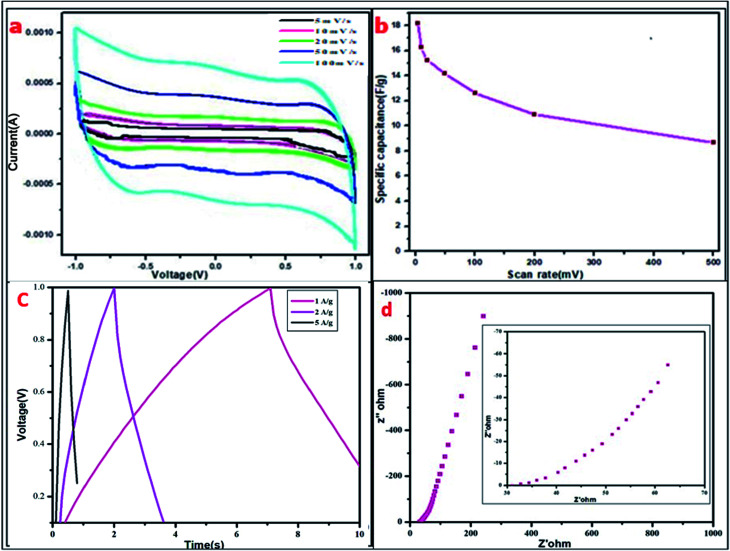
(a) CV analysis with gel electrolyte of PVA–H_3_PO_4_, (b) variation in the specific capacitance with scan rate, (c) GCD analysis in gel electrolyte of PVA–H_3_PO_4_, and (d) electrochemical impedance spectroscopy for PVA–H_3_PO_4_ gel electrolyte.

Further, the GCD curve was plotted for voltage *vs.* time (s) for the fabricated device (Fig. 6). The GCD curve showed slightly distorted CD curves, which may be due to faradaic reactions during the charge storage mechanism of supercapacitors. Here, the device operated under the current densities of 1, 2, and 5 A g^−1^ having the potential opening of 0–1 V. The relative specific capacitance was measured using following the expression (eqn (4)):4
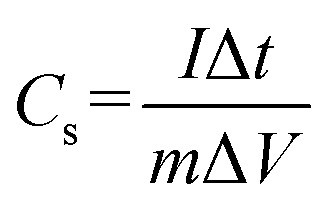
where *C*_s_ represents the specific capacitance of the device in F g^−1^, *I* is the discharge current in A, Δ*V* represents the potential window in volts, and *m* is the active mass of material in mg. The fabricated device showed a specific capacitance of 14.25 F g^−1^ at the current density of 1 A g^−1^. Furthermore, the specific capacitance values for the fabricated device at varying current densities are presented in Table 5.

**Table 5:** The specific capacitance values based on the GCD and EIS data

Current density	1 A g^−1^	2 A g^−1^	5 A g^−1^
Specific capacitance (F g^−1^) through GCD	14.25	7.408	5.76
Specific capacitance (F g^−1^) through EIS at 10 mHz	12.94		

### 3.4. Electrochemical impedance spectroscopy

The electrochemical impedance spectroscopy (EIS) data was evaluated, and the Nyquist plot is shown in Fig. 6d for the fabricated device in the frequency range of 10 mHz to 106 Hz. EIS spectroscopy is mainly used for the detection of ionic transportation and the electronic behavior of electrode materials. EIS data can be pretty well shown in the complex plane, which is called the Nyquist plot, where the real part is represented as *Z*′, and the imaginary part as *Z*′′ for the impedance was plotted in this plane. The real part (*Z*′) lies on the *x*-axis, while the imaginary part (*Z*′′) lies on the *y*-axis. The resulting plot is a hodograph, *i.e.*, having the frequency as the crucial parameter. An ideal capacitor shows the equivalent series resistance (ESR) as a straight shift in the vertical line on the *x* axis with respect to frequency. As shown in Fig. 6d, the supercapacitor performance was distributed in two regions, one at higher frequencies, where this portion of the graph corresponds to the diffusion process, which is related to the ability of ions to penetrate the pores, as modeled by a distributed resistance together with a distributed capacitance.^30–32^ Conversely, the vertical line of the same plot in the lower frequency region is related to the capacitive performance. The continuous EIS evaluations at higher frequencies resulted in a lowering of the imaginary region (*Z*′′) down to the *x*-axis, relating to very inductive behaviour and can be viewed as series inductance, which is responsible for the diffracted buildup of the structure.^32–34^ The EIS resistance can be related to the resistances of the polymer gel-electrolyte together with the positive and negative electrodes.

Further the EIS-based capacitance value was calculated using following relation:
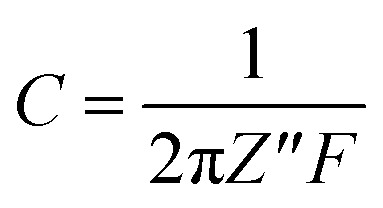
where *Z*′′ is the maximum value of the *Y* axis, *F* is the frequency used and *C* is the capacitance.

**Fig. 7:**
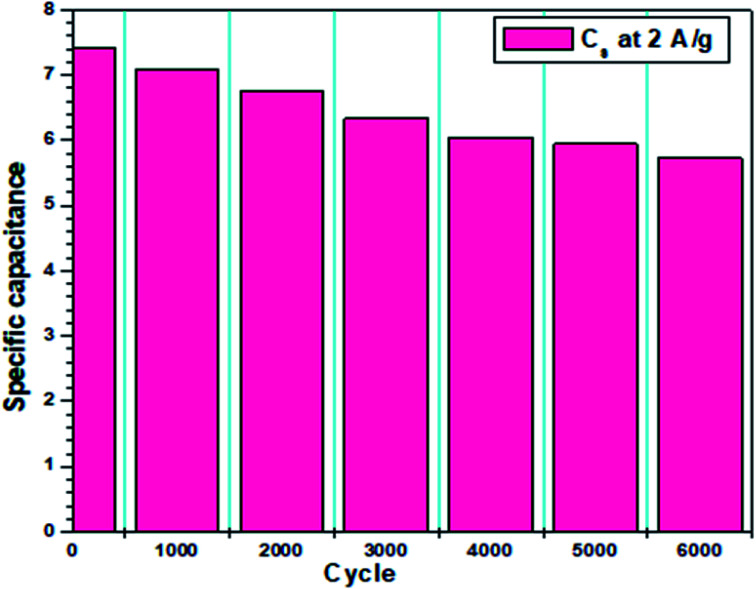
Variation in the specific capacitance up to 3000 cycles.

The corresponding EIS plot showed a specific capacitance of 12.94 F g^−1^ (Table 5). Here, the linear nature of the EIS plot around the low-frequency range indicates the resistance of the electrolyte ions during the ionic transportation over other side of the double layer.^33–35^ The cyclic stability of the fabricated device is shown in Fig. 7, which was measured for more than 3000 cycles *via* the charge–discharge mechanism. The current density of 2 A g^−1^ was used for the detection of cyclic stability, and 85% retention was found for the initial 1500 cycles from the initial capacitance value, and overall, 78% retention for 3000 cycles.

## 4. Conclusion

Herein, we reported the mass-scale production of MDGs, where we successfully produced 2.0 kg of MDGs using 5 kg waste materials, *i.e.*, *Quercus ilex* leaves, as the precursor material. The Raman, XRD, FT-IR, and SEM/EDX data completely identified the material as MDGs. The *in situ* quantity of potassium was found to be 6.15%. The production of MDGs was done through a very eco-friendly and cost-effective procedure with almost no harmful emissions. This is very innovative modelling of agricultural waste into advanced materials, which not only advances this technology in various fields, but also suggests innovations to be more cost-effective and eco-friendly with less hazards to the environment. The theoretical and computational DFT study confirmed the positioning of the dopants in the MDG sample follows the chemisorption/decoration pattern, substantially as van der Waals interactions with the graphene nanosheets, rather than the substitutional pattern. The synthesized MDGs were further used for the fabrication of a supercapacitor device, consisting of PVA–H_3_PO_4_ as a polymer gel electrolyte. A maximum specific capacitance of 18.2 F g^−1^ was reported at the scan rate of 5 mV s^−1^ using PVA–H_3_PO_4_ as the electrolyte.

The CV data showed a high power density of 1000 W kg^−1^ at 5 A g^−1^ and 85% capacitance retention was found after 1500 cycles from the initial capacitance value. The reported MDG material can also act as an excellent candidate for futuristic growth and development in various fields, such as energy storage and energy conservation applications, water purification, and bio-imaging, with the sufficiently advanced and progressive mass-scale production of MDGs.

## Conflicts of interest

There are no conflicts to declare.

## Supplementary Material
